# A non-canonical role of *ELN* protects from cellular senescence by limiting iron-dependent regulation of gene expression

**DOI:** 10.1016/j.redox.2024.103204

**Published:** 2024-05-22

**Authors:** Joanna Czarnecka-Herok, Kexin Zhu, Jean-Michel Flaman, Delphine Goehrig, Mathieu Vernier, Gabriela Makulyte, Aline Lamboux, Helena Dragic, Muriel Rhinn, Jean-Jacques Médard, Gilles Faury, Philippe Bertolino, Vincent Balter, Romain Debret, Serge Adnot, Nadine Martin, David Bernard

**Affiliations:** a Centre de Recherche en Cancérologie de Lyon, Inserm U1052, CNRS UMR 5286, Centre Léon Bérard, Université de Lyon, Lyon, France; b Equipe Labellisée la Ligue Contre le Cancer, Lyon, France; c Laboratoire de Géologie de Lyon: Terre, Planètes, Environnements, CNRS UMR 5276, Ecole Normale supérieure de Lyon, Lyon, France; d Institut de Génétique et de Biologie Moléculaire et Cellulaire, CNRS UMR7104, Inserm U1258, Université de Strasbourg, Illkirch, 67404, France; e Université Grenoble Alpes, Inserm, CHU Grenoble Alpes, HP2, Inserm U1300, 38000, Grenoble, France; f Laboratoire de Biologie Tissulaire et Ingénierie Thérapeutique, UMR5305 CNRS/ Université Claude Bernard Lyon 1, 7 passage du Vercors, 69007, Lyon, France; g Inserm U955, Département de Physiologie - Explorations fonctionnelles, Hôpital Henri Mondor, AP-HP, FHU SENEC, Créteil, France; h Institute of Lung Health, Justus Liebig University, Giessen, Germany

**Keywords:** Cellular senescence, ELN, HMOX1, ROS, Iron, PHF8

## Abstract

The *ELN* gene encodes tropoelastin which is used to generate elastic fibers that insure proper tissue elasticity. Decreased amounts of elastic fibers and/or accumulation of bioactive products of their cleavage, named elastokines, are thought to contribute to aging. Cellular senescence, characterized by a stable proliferation arrest and by the senescence-associated secretory phenotype (SASP), increases with aging, fostering the onset and progression of age-related diseases and overall aging, and has so far never been linked with elastin. Here, we identified that decrease in *ELN* either by siRNA in normal human fibroblasts or by knockout in mouse embryonic fibroblasts results in premature senescence. Surprisingly this effect is independent of elastic fiber degradation or elastokines production, but it relies on the rapid increase in HMOX1 after *ELN* downregulation. Moreover, the induction of HMOX1 depends on p53 and NRF2 transcription factors, and leads to an increase in iron, further mediating *ELN* downregulation-induced senescence. Screening of iron-dependent DNA and histones demethylases revealed a role for histone PHF8 demethylase in mediating *ELN* downregulation-induced senescence. Collectively, these results unveil a role for *ELN* in protecting cells from cellular senescence through a non-canonical mechanism involving a ROS/HMOX1/iron accumulation/PHF8 histone demethylase pathway reprogramming in gene expression towards a senescence program.

## 1 Introduction

Elastin is a major extracellular matrix polymer and is widely recognized for its unique elastic properties. Together with fibrillin-rich microfibrills, elastin forms a backbone of elastic fibers and in this form provides stretch and recoil for many organs including the skin, blood vessels and lungs. Elastin is encoded by the *ELN* gene and is synthetized as a soluble monomer called tropoelastin, which after secretion undergoes post-translational modifications in an elaborate process of elastic fiber maturation [1,2]**.** *ELN* expression primarily occurs during early development and tropoelastin production decreases after reaching organism maturity. During the aging process, deterioration and decrease in elastic fibers are observed, which can contribute to age-related diseases by perturbing elasticity of multiple tissues. For instance, its decrease during aging impacts several tissues resulting in increased risks of developing cardiovascular diseases, chronic obstructive pulmonary disease and osteoarthritis. Thus decreased levels of elastic fibers, including through decreased *ELN* expression, can significantly impact tissue health and function during aging [[3], [4], [5], [6]]. Aside from its effect on tissue elasticity, breakdown of elastic fibers during aging releases small bioactive peptides called elastokines (EDP – elastin-derived peptides), which were reported to stimulate angiogenesis, regulate cell adhesion, migration, proliferation, proteolytic activity and apoptosis and to promote some age-related diseases (reviewed in [7]).

It is well established that senescent cells contribute to overall aging of organisms and age-related pathologies [[8], [9], [10]]. Cellular senescence is a state of stable proliferation arrest that is caused by various stimuli, including telomere shortening and oxidative or oncogenic stresses. Senescent cells are characterized by stable cell cycle arrest, increased levels of SA-β-galactosidase activity, production of high levels of reactive oxygen species (ROS), decreased DNA repair, gene expression changes, epigenetic modifications and abundant secretion of numerous factors globally referred to as the senescence-associated secretory phenotype (SASP) [11]. Mechanisms controlling cellular senescence are still being elucidated, however the main master regulators known to date regulate either cell cycle progression or SASP, namely cyclin-dependent kinase inhibitors such as p21/CDKN1A, p16/CDKN2A, Retinoblastoma (RB) and p53 proteins or mTOR, NFκB and C/EBPβ [11]. Whether *ELN* can regulate cellular senescence is currently unknown.

Here we discovered an unexpected function for *ELN* in regulating cellular senescence and uncovered an original mechanism of action relying on heme signaling and regulation of an iron-dependent gene expression, likely independent of *ELN* known canonical functions.

## 2 Results

### 2.1 ELN downregulation triggers cellular senescence

We initially interrogated different datasets to determine whether *ELN* was differentially expressed during cellular senescence. Strikingly, *ELN* mRNA levels were lower during cellular senescence in different types of cells and following different pro-senescence stresses including telomere shortening or oncogenic insult (Table 1).

**Table 1:** **Transcriptomic datasets showing downregulation of ELN levels in different models of senescence.** Senescence was induced by hydrogen peroxide (H2O2), RAS oncogene or telomere shortening (replicative senescence, RS) in the indicated cells.

	Astrocytes H2O2 (GSE58910)	IMR90 RAS (GSE60652)	MRC5 RS (GSE15919)	MEF RAS (GSE210060)
Fold down-regulation of ELN in senescence	6.7	3.12	2.25	25

**Fig. 1:**
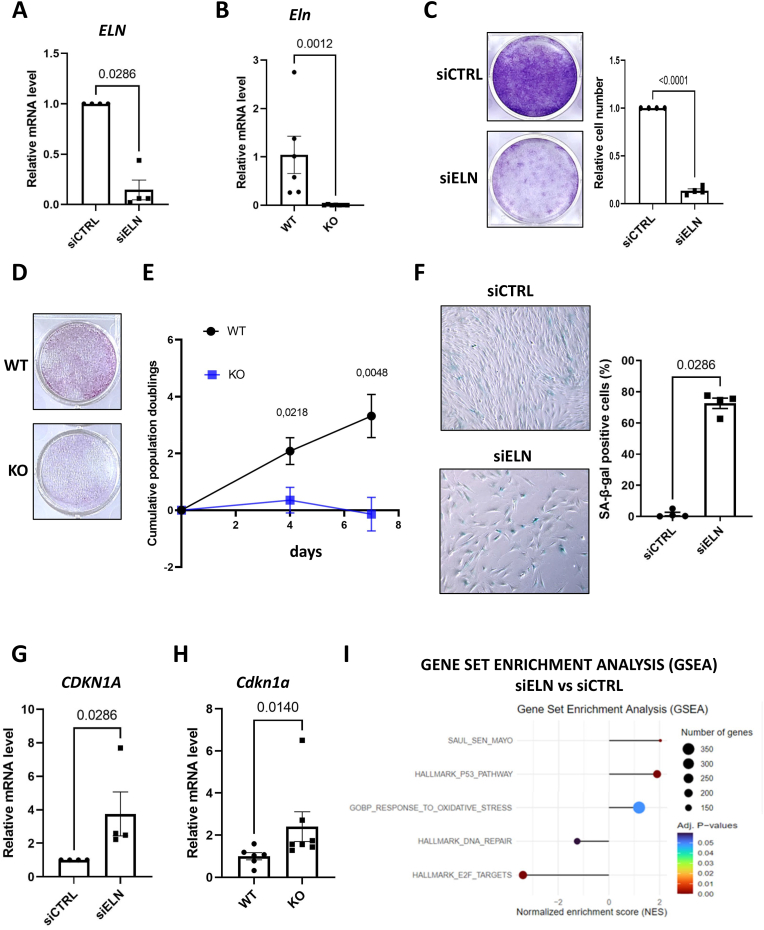
*ELN* downregulation triggers premature senescence. **A.** RT-qPCR of *ELN* gene at day 4 after transfection of MRC5 human fibroblasts with a non-targeting siRNA pool (siCTRL) or a siRNA pool targeting ELN (siELN). Mean ± SEM of n = 4 independent experiments. Two-tailed Mann-Whitney *U* Test. **B**. RT-qPCR of *Eln* gene in wild type (WT) and *Eln* knock-out (KO) mouse embryonic fibroblasts 4 days after plating. Mean ± SEM of n = 6–10 independent embryos. Two-tailed Mann-Whitney *U* Test. **C**. Crystal violet staining (left panel) and quantification of cell number (right panel) at day 7 after transfection of MRC5 human fibroblasts with siCTRL or siELN. Left panel: representative experiment (n = 4). Right panel: mean ± SEM of n = 4 independent experiments, unpaired two-tailed Welch's *t*-test. **D**. Crystal violet staining of WT and *Eln* KO mouse embryonic fibroblasts 7 days after plating. Representative experiment (n = 5). **E**. Growth curves of WT and *Eln* KO mouse embryonic fibroblasts. Mean ± SEM of n = 6–7 independent embryos. Unpaired two-tailed Welch's *t*-test. **F**. Representative micrographs (left panel) and quantification (right panel) of SA-β-galactosidase (SA-β-gal) positive cells in MRC5 human fibroblasts at day 4 after transfection with siCTRL or siELN. Mean ± SEM of n = 4 independent experiments. Two-tailed Mann-Whitney *U* Test. **G**. RT-qPCR of *CDKN1A* gene at day 4 after transfection of siCTRL or siELN in MRC5 human fibroblasts. Mean ± SEM of n = 4 independent experiments. Two-tailed Mann-Whitney *U* Test. **H**. RT-qPCR of *Cdkn1a* gene in WT and *Eln* KO mouse embryonic fibroblasts at day 4 after plating. Mean ± SEM of n = 6–7 independent embryos. Two-tailed Mann-Whitney *U* Test. **I**. Enrichment of senescence-associated gene sets in transcriptomes of MRC5 4 days after transfection with siELN *versus* siCTRL (n = 3), according to gene set enrichment analysis (GSEA).

To investigate the impact of *ELN* on cell proliferation, we studied the effects of its downregulation in MRC5 human lung fibroblasts. We first confirmed *ELN* downregulation after siRNA transfection using a pool of 4 siRNA (Fig. 1A) or two individual siRNA (Suppl. Fig. 1A). In parallel, we studied mouse embryonic fibroblasts (MEFs) either wild type or harboring an *Eln* knock-out (KO) (Fig. 1B). Strikingly, *ELN* loss strongly reduced the proliferative capacity of MRC5 cells (Fig. 1C, Suppl. Fig. 1B) and of MEFs (Fig. 1D–E). This decrease in cell population during *ELN* knockdown in MRC5 or *Eln* knockout in MEFs was accompanied by other marks of cellular senescence: (i) an increased proportion of SA-β-galactosidase-positive cells (Fig. 1F and Suppl. 1C, D), (ii) an increased mRNA level of the cyclin-dependent kinase inhibitor *CDKN1A* (Fig. 1G–H and Suppl. Fig. 1E), and (iii) an increased level of SASP factors as evidenced by increased mRNA levels of *GDF15*, *MMP3*, *BMP2* and *ANGPTL4* (Suppl. Fig. 1F).

Whole-genome transcriptome analysis performed 4 days after siRNA transfection identified about 2000 differentially expressed genes (DEGs) upon *ELN* downregulation (Suppl. Fig. 1G) and Geneset Enrichment Analysis (GSEA) revealed a positive enrichment of several gene sets associated with cellular senescence. Senescence-associated signatures, for instance, SASP (SAUL_SEN_MAYO), p53 pathway or response to oxidative stress, were significantly higher in ELN-depleted cells, whereas signatures related to DNA repair and E2F targets were lower, consistent with a senescent phenotype (Fig. 1I).

Of note, in MRC5 cells, no elastin fibers could be detected by immunofluorescence on a confluent culture, although elastin fibers were visible on a confluent culture of normal human dermal fibroblasts (NHDF) stained in parallel (Suppl. Fig. 1H), ruling out an elastin fiber-dependent effect of *ELN* knockdown in MRC5 cells.

Together, these results support that *ELN* downregulation induces premature senescence independently of its role in elastin fibers.

### 2.2 Heme oxygenase 1 participates in ELN downregulation-induced senescence

**Fig. 2:**
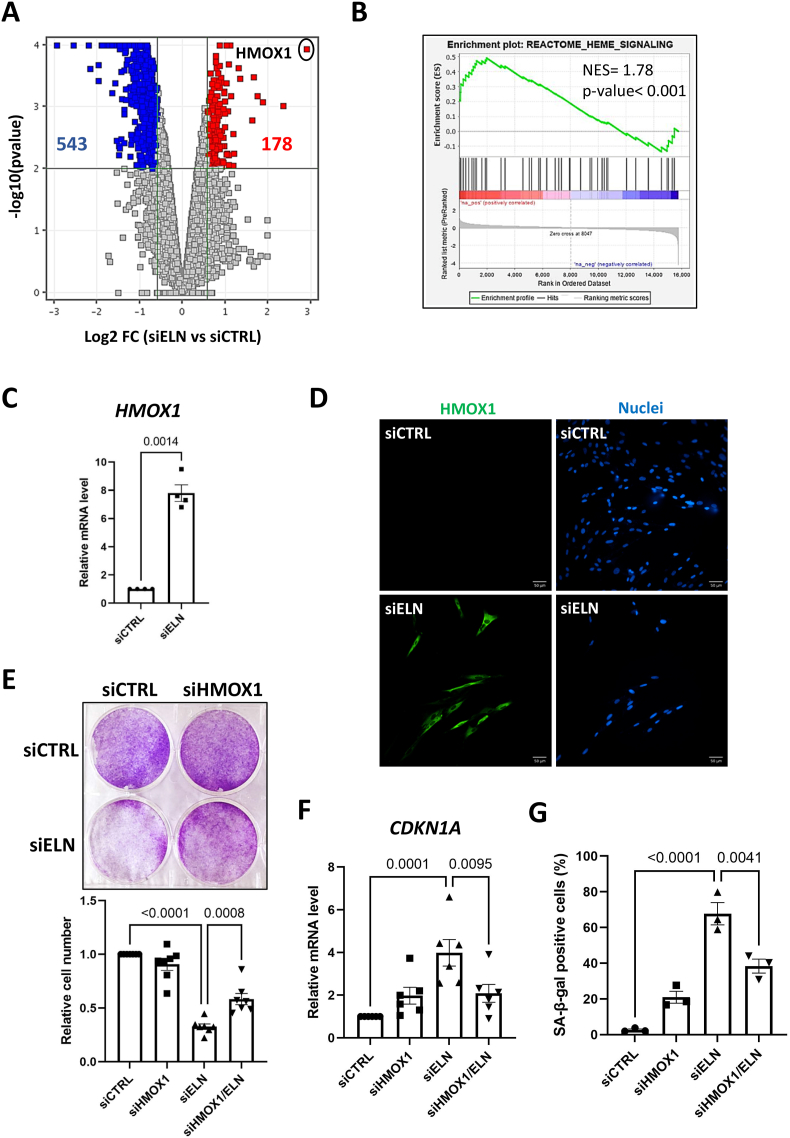
HMOX1 mediates senescence induced by *ELN* downregulation. **A**. Volcano plot showing differentially expressed genes in siELN *versus* siCTRL MRC5 cells 1 day after siRNA transfection, according to transcriptomic analysis (n = 3). **B**. Enrichment of heme signaling gene set in MRC5 cells 1 day after transfection with siELN *versus* siCTRL, according to transcriptomic analysis (n = 3) followed by gene set enrichment analysis (GSEA). **C**. RT-qPCR of *HMOX1* gene one day after transfection of MRC5 cells with siCTRL or siELN. Mean ± SEM of n = 4 independent experiments. Unpaired two-tailed Welch's *t*-test. **D**. Immunofluorescence micrographs of MRC5 cells 3 days after transfection with siCTRL or siELN, showing staining of HMOX1 protein (green) and of nuclei with Hoechst 33,342 (blue) (representative pictures of n = 3 independent experiments). **E**. Crystal violet staining (top panel) and quantification of cell number (lower panel) 7 days after transfection of MRC5 cells with siCTRL, siELN and/or siHMOX1 as indicated. Top panel: representative experiment (n = 4). Lower panel: mean ± SEM of n = 7 independent experiments, one-way ANOVA, Tukey's multiple comparisons test. **F**. RT-qPCR of *CDKN1A* gene at day 4 after transfection of MRC5 cells with siCTRL, siELN and/or siHMOX1 as indicated. Mean ± SEM of n = 6 independent experiments. One-way ANOVA. Tukey's multiple comparisons test. **G**. Quantification of SA-β-galactosidase (SA-β-gal) positive cells 4 days after transfection of MRC5 cells with siCTRL, siELN and/or siHMOX1 as indicated. Mean ± SEM of n = 3 independent experiments. One-way ANOVA. Tukey's multiple comparisons test.

To elucidate the mechanisms underlying induction of senescence by decreasing *ELN*, we performed transcriptome analysis at an earlier time point than the one used for characterizing the senescent phenotype. Interestingly, transcriptome analysis performed 24 h after *ELN* knockdown revealed 721 DEGs (Fig. 2A). GSEA analysis revealed a positive enrichment of heme signaling genes (Fig. 2B, Suppl. Fig. 2A). Alterations in heme synthesis and degradation have been linked with aging, especially heme degradation, which has been shown to increase with age and after exposure to stress [12,13]. Heme oxygenase 1, HMOX1, was identified as the most upregulated gene at this early time point (Fig. 2A). HMOX1 is an inducible enzyme degrading heme and was reported to be induced during cellular senescence in several contexts, with complex and sometimes opposite effects as it is has been described to have mostly anti-senescence or pro-senescence effects pending of the contexts [[14], [15], [16], [17]]. In addition, increased levels of HMOX1 are known to induce mitochondrial oxidative stress [18]. *ELN* downregulation led to a strong increase in HMOX1 both at mRNA, confirming transcriptomic data, and protein levels (Fig. 2C and D). We thus sought to determine whether lowering HMOX1 would impact senescence induced by *ELN* downregulation. To test this, MRC5 cells were transfected with siRNA to knock down *ELN* and *HMOX1* (Suppl. Fig. 2B). Decreasing *HMOX1* levels impaired the induction of senescence by *ELN* downregulation, as evidenced by rescue of MRC5 proliferation (Fig. 2E), the decrease in the cyclin-dependent kinase inhibitor *CDKN1A* mRNA level (Fig. 2F) and the decrease in the proportion of SA-β-galactosidase-positive cells (Fig. 2G). Nevertheless, decrease of HMOX1 alone slightly increased *CDKN1A* mRNA levels and SA-β-galactosidase-positive cells (Fig. 2F–G). Hence, the rapid induction of HMOX1 during loss of ELN fosters premature senescence.

### 2.3 Heme oxygenase 1 is upregulated through TP53, NRF2 and ROS

**Fig. 3:**
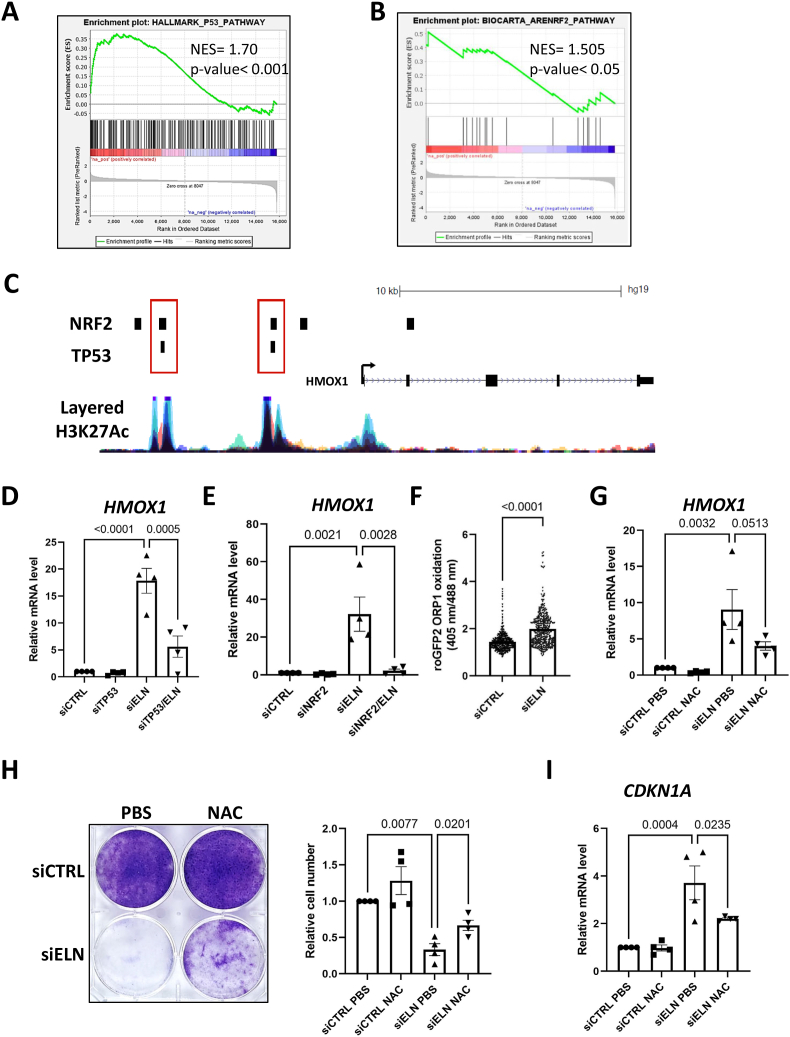
TP53, NRF2 and ROS participate in the induction of *HMOX1* expression upon *ELN* downregulation. **A-B**. Enrichment of p53 (A) and NRF2 (B) pathways gene sets in MRC5 cells 1 day after transfection with siELN *versus* siCTRL, according to transcriptomic analysis (n = 3) and GSEA. **C**. ChIP-seq profiles of NRF2 (NFE2L2 ChIP-seq GSE91894) and TP53 (TP53 ChIP-seq GSE100292). The track for H3K27Ac is the overlay of 7 different chip-seq (default representation on UCSC). **D**. RT-qPCR of *HMOX1* gene at day 2 after transfection of MRC5 cells with siCTRL, siELN and/or siTP53 as indicated. Mean ± SEM of n = 4 independent experiments. One-way ANOVA. Tukey's multiple comparisons test. **E**. RT-qPCR of *HMOX1* gene at day 2 after transfection of MRC5 cells with siCTRL, siELN and/or siNRF2 as indicated. Mean ± SEM of n = 3 independent experiments. One-way ANOVA. Tukey's multiple comparisons test. **F**. Single-cell analysis of mitochondrial ROS with the roGFP2 ORP1 genetic probe one day after transfection of MRC5 cells with siCTRL and siELN. N = 4 independent experiments, n = 6047 analyzed cells for siCTRL and n = 6116 analyzed cells for siELN. Two-tailed Mann-Whitney *U* Test. **G**. RT-qPCR of *HMOX1* gene one day after transfection of MRC5 cells with siCTRL or siELN and 1 mM NAC treatment where indicated. Mean ± SEM of n = 4 independent experiments. One-way ANOVA. Tukey's multiple comparisons test. **H**. Crystal violet staining (left panel) and quantification of cell number (right panel) 7 days after transfection of MRC5 cells with siCTRL or siELN and one single treatment with 1 mM N-acetyl cysteine (NAC) antioxidant or PBS as control. Left panel: representative experiment (n = 4). Right panel: mean ± SEM of n = 4 independent experiments, one-way ANOVA, Tukey's multiple comparisons test. **I**. RT-qPCR of *CDKN1A* gene at day 4 after transfection of MRC5 cells with siCTRL or siELN and 1 mM NAC treatment where indicated. Mean ± SEM of n = 4 independent experiments. One-way ANOVA. Tukey's multiple comparisons test.

This sharp and rapid induction of HMOX1 after *ELN* knockdown once again supports an elastic fiber-independent effect and reveals an unsuspected function for ELN. In line with the fact that TP53 and NRF2 transcription factors are known activators of *HMOX1* transcription [[19], [20], [21]], TP53 and NRF2 pathway activation were observed the day after transfection with siRNA against *ELN* (Fig. 3A–B). Analysis of publicly available ChIP-seq data revealed that TP53 and NRF2 could bind 2 common regions of the *HMOX1* promoter and that these regions are enriched in H3K27Ac histone mark, which is associated with activation of transcription (Fig. 3C). These results suggest that NRF2 and TP53 co-activate *HMOX1* expression during senescence induced by ELN loss. To test this hypothesis MRC5 cells were transfected with siRNA directed against *ELN* and either *TP53* or *NRF2*. We initially confirmed the knockdown of *ELN* and *TP53* or *NRF2* (Suppl. Figs. 3A and B). Both *TP53* and *NRF2* downregulation led to a strong decrease in *HMOX1* mRNA upregulation in siELN-transfected cells (Fig. 3D and E). The impact of TP53 knockdown on the number of cells (Suppl. Fig. 3C) and on the proportion of SA-β-galactosidase-positive cells (Suppl. Fig. 3D) indicated that TP53 plays a functional role in senescence induced by *ELN* downregulation. *NRF2* knockdown induced similar partial rescue of this phenotype, as illustrated by the number of cells (Suppl. Fig. 3E) and the proportion of SA-β-galactosidase-positive cells (Suppl. Fig. 3F).

In addition, the activation of the NRF2-HMOX1 axis and the above determined upregulation of the oxidative stress response genes (Fig. 1I) suggest the possible implication of reactive oxygen species (ROS), which also impact the p53 pathway [22], in underlying mechanisms. Using roGFP2-ORP-1 ratiometric mitochondrial ROS sensor, we observed a significant increase in ROS following *ELN* downregulation (Fig. 3F). We then determined whether scavenging ROS would impact *HMOX1* upregulation and senescence induced by *ELN* downregulation. Functional tests using the potent antioxidant N-acetyl cysteine (NAC) upon *ELN* knockdown (Suppl. Fig. 3G) showed partial rescue of *HMOX1* mRNA levels (Fig. 3G), of MRC5 proliferation (Fig. 3H) and of *CDKN1A* expression (Fig. 3I).

Collectively, these data unveiled that HMOX1 is upregulated through TP53, NRF2 and ROS in response to *ELN* downregulation.

### 2.4 Iron promotes ELN downregulation-induced senescence

**Fig. 4:**
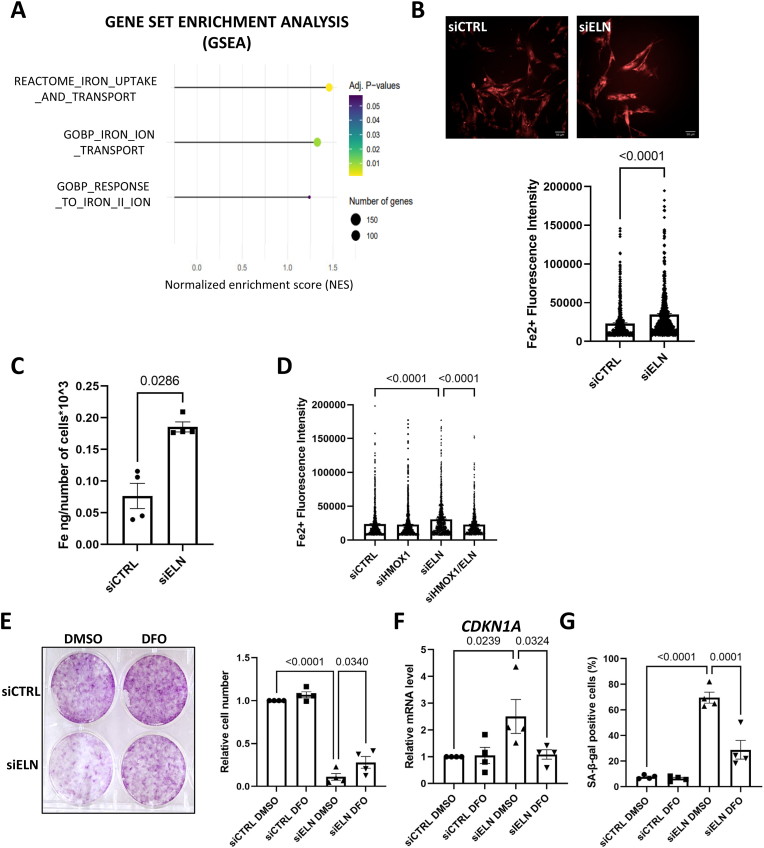
Iron plays a significant role in senescence induced by *ELN* downregulation. **A**. Enrichment of iron-related gene sets in transcriptomes of MRC5 human fibroblasts 4 days after transfection with siELN *versus* siCTRL (n = 3), according to gene set enrichment analysis (GSEA). **B**. Representative micrographs (left panel) and single-cell quantification (right panel) of staining with SiRhoNox-1, a fluorescent probe labeling Fe^2+^, 2 days after transfection of MRC5 cells with siCTRL or siELN. N = 3 independent experiments, n = 622 analyzed cells for siCTRL and n = 615 analyzed cells for siELN. Two-tailed Mann-Whitney *U* Test. **C**. Total iron measurement by ICP-MS at day 2 after transfection of MRC5 cells with siCTRL or siELN. Mean ± SEM of n = 4 independent experiments. Two-tailed Mann–Whitney *U* Test. **D**. Single-cell quantification of staining with SiRhoNox-1, 2 days after transfection of MRC5 cells with siCTRL, siELN and/or siHMOX1. N = 3 independent experiments, n = 1033 analyzed cells for siCTRL, n = 1015 analyzed cells for siHMOX1, n = 1042 analyzed cells for siELN, n = 1012 analyzed cells for siELN/HMOX1 in total. One-way ANOVA, Tukey's multiple comparisons test. **E**. Crystal violet staining (left panel) and quantification of cell number (right panel) 7 days after transfection of MRC5 cells with siCTRL or siELN and treatment with 72 nM deferoxamine (DFO) iron chelator or DMSO (as control). Left panel: representative experiment (n = 3). Right panel: mean ± SEM of n = 4 independent experiments, one-way ANOVA, Tukey's multiple comparisons test. **F**. RT-qPCR of *CDKN1A* gene at day 4 after transfection of MRC5 cells with siCTRL or siELN and treatment with DFO where indicated. Mean ± SEM of n = 4 independent experiments. One-way ANOVA. Tukey's multiple comparisons test. **G**. Quantification of SA-β-gal-positive cells at day 4 after transfection of MRC5 cells with siCTRL or siELN and treatment with DFO where indicated. Mean ± SEM of n = 4 independent experiments. One-way ANOVA. Tukey's multiple comparisons test.

HMOX1 is an inducible enzyme that catabolizes heme degradation into carbon monoxide, biliverdin and labile iron (Fe^2+^). Interestingly, whole-genome transcriptome analysis revealed positive enrichment of genes involved in iron uptake and transport, iron ion transport and response to iron II ion (Fig. 4A). Given that HMOX1 increases during aging, that age-related pathologies [23] have been associated with accumulation of iron [24,25], and that iron has also been shown to accumulate with aging *in vivo* [[26], [27], [28], [29]] and *in vitro* [30,31], we wondered whether iron accumulation could contribute to cellular senescence. Increase in ferrous iron was confirmed following *ELN* downregulation using the SiRhoNox-1 fluorescence probe (Fig. 4B); such an increase in labile iron has already been shown in senescent cells with the same probe [32,33]. Not only was labile iron increased but also total iron levels, as evidenced by ICP-MS (inductively coupled plasma mass spectrometry) (Fig. 4C). As expected, the increase in iron during *ELN* downregulation was dependent on HMOX1 (Fig. 4D). Next, to assess whether iron plays a functional role in senescence induced by *ELN* downregulation, iron was chelated by using the iron chelator deferoxamine (DFO). Iron chelation led to partial rescue of cell proliferation (Fig. 4E), lowered *CDKN1A* mRNA levels (Fig. 4F) and the proportion of SA-β-galactosidase-positive cells (Fig. 4G) in response to *ELN* knockdown (Suppl. Fig. 4).

Taken together, these data establish a direct functional link between HMOX1 upregulation, iron increase and cellular senescence induced by *ELN* downregulation.

### 2.5 ELN downregulation drives changes in gene expression through the iron-dependent demethylase PHF8

**Fig. 5:**
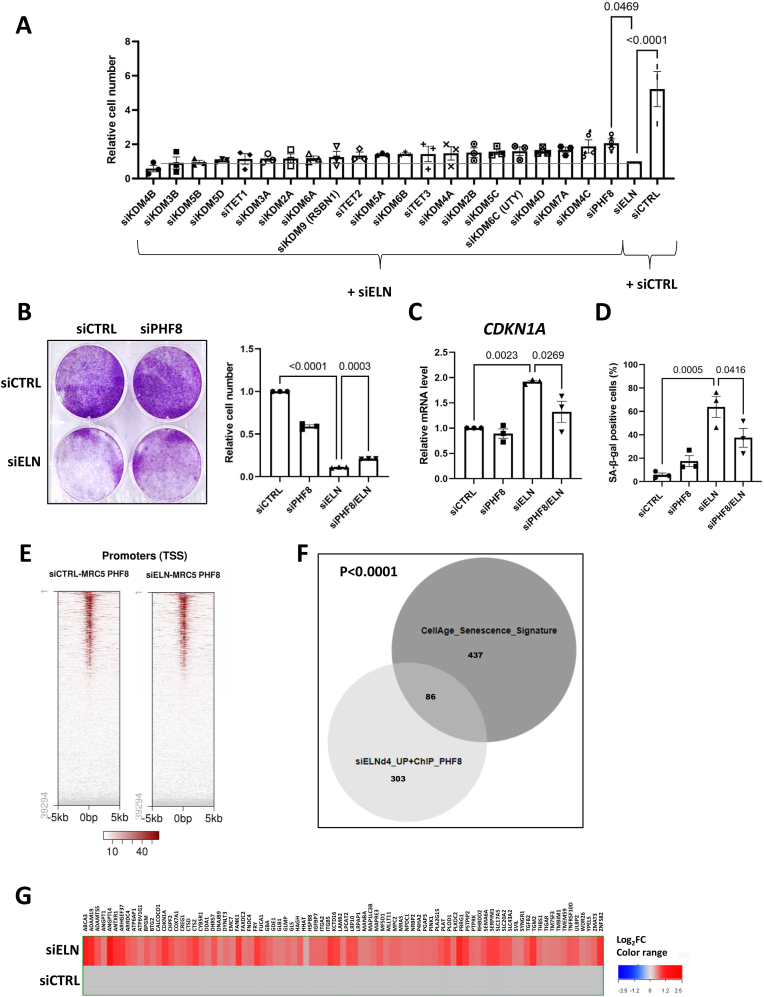
The iron-dependent demethylase PHF8 contributes to senescence induction upon *ELN* downregulation. **A**. Quantification of cell number 7 days after transfection of MRC5 cells with the indicated siRNAs. Mean ± SEM of n = 3 independent experiments. One-way ANOVA. Tukey's multiple comparisons test. **B**. Crystal violet staining (left panel) and quantification of cell number (right panel) 7 days after siRNA transfection of MRC5 cells with siCTRL, siELN and/or siPHF8. Left panel: representative experiment (n = 3). Right panel: mean ± SEM of n = 3 independent experiments, one-way ANOVA, Tukey's multiple comparisons test. **C**. RT-qPCR of *CDKN1A* gene at day 4 after transfection of MRC5 cells with siCTRL, siELN and/or siPHF8. Mean ± SEM of n = 3 independent experiments. One-way ANOVA. Tukey's multiple comparisons test. **D**. Quantification of SA-β-gal-positive cells at day 4 after transfection of MRC5 cells with siCTRL, siELN and/or siPHF8. Mean ± SEM of n = 3 independent experiments. One-way ANOVA. Tukey's multiple comparisons test. **E**. Heatmaps showing peaks of PH8 on promoter regions in siCTRL- and siELN-transfected MRC5. **F**. Venn diagram showing the number of genes in common between genes bound by PHF8 and up-regulated by ELN knockdown (4 days after siRNA transfection), and genes of the CellAge signature. Fisher's exact test P value is indicated for CellAge signature enrichment. **G.** Heatmap showing the profile of expression of the 89 genes that intersect in panel F according to the transcriptome data 4 days after transfection of siELN vs siCTRL.

One of the roles of iron is to act as cofactor for several oxygenases regulating the epigenome, including several iron-dependent histone and DNA demethylases [34,35]. Interestingly, some of these enzymes are known regulators of cellular senescence [[36], [37], [38]]. We performed siRNA screening to test the involvement of 21 iron-dependent oxygenases in the induction of *ELN* downregulation-induced senescence. Among these, we identified the iron-dependent histone demethylase plant homeodomain finger protein 8 (PHF8) as a factor significantly promoting proliferation arrest following *ELN* downregulation (Fig. 5A). PHF8 is known to promote transcription by demethylating histone repressive marks [39,40], though its role in regulating cellular senescence has not yet been reported. We confirmed that *PHF8* downregulation (Suppl. Fig. 5A) led to partial rescue of cell proliferation (Fig. 5B) upon ELN knockdown (Suppl. Fig. 5A), and observed that it lowered *CDKN1A* mRNA levels (Fig. 5C) and the proportion of SA-β-galactosidase-positive cells (Fig. 5D), strongly supporting its role in mediating *ELN* downregulation-induced senescence. ChIP-seq analysis against PHF8 revealed its binding to the promoter of approximately 12,000 genes, and this binding profile was not altered by *ELN* knockdown (Fig. 5E and Suppl. Figs. 5B–C). A significant number of genes showed both an increased expression upon *ELN* downregulation and a binding of PHF8 to their promoter: 93 at day 1, and 389 at day 4 (Suppl. Table 1). Crossing these lists of genes at day 1 and day 4 with a cellular senescence signature based on the CellAge database revealed a strong enrichment in genes involved in cellular senescence at day 4 including *CDKN1A* and SASP factors (Fig. 5F–G), and not at day 1 (Suppl. Fig. 5D), further supporting that iron-bound PHF8 contributes to the regulation of a large panel of pro-senescent genes during *ELN* downregulation-induced senescence.

Together, these data indicate that iron-dependent demethylase PHF8 and its transcriptional program, probably downstream of iron accumulation, are important players in the induction of senescence upon *ELN* downregulation.

## 3 Discussion

We herein demonstrated for the first time that *ELN* downregulation in human fibroblasts and *Eln* knockout in MEFs trigger premature cellular senescence. Mechanistically, *ELN* downregulation leads to ROS, p53 and NRF2-dependent increase in HMOX1 expression which causes iron release. We further identified a role for the iron-dependent PHF8 demethylase in the upregulation of multiple pro-senescent genes and in inducing senescence upon *ELN* downregulation.

Elastin fibers can impact health by regulating tissue elasticity and/or by releasing bioactive elastokine peptides during elastin fiber cleavage by proteases [41,42], which is thought to promote several age-related diseases [[43], [44], [45], [46]]. As senescent cells promote age-related diseases and aging [[8], [9], [10]], a raising question is whether elastin regulates cellular senescence. Our results obtained by knocking-down *ELN* in human fibroblasts or by using fibroblasts derived from *Eln* −/− embryos demonstrate that decreasing *ELN* expression triggers premature senescence.

Some evidence supports that reduced levels of *ELN* can be linked to accelerated aging. Williams-Beuren syndrome (WBS) is a developmental disorder caused by the hemizygous deletion of a region containing 26 to 28 genes comprising *ELN* on chromosome 7 and characterized by an array of features including intellectual disability, cardiovascular disease, and additional symptoms in the adulthood such as diabetes, premature aging of the skin and osteoporosis [5], which led to the hypothesis of mild accelerated aging [6]. Heterozygous deletion of the WBS critical region in mice was shown to induce premature cell proliferation arrest in MEFs [47], suggesting premature senescence. *ELN* hemizygous loss is considered to be the main trigger of cardiovascular pathology in WBS and is also proposed to contribute to the soft skin observed in WBS patients [5]. Our results suggest that reduced *ELN* levels could promote WBS features through the induction of cellular senescence.

In addition to showing a protective role for *ELN* against cellular senescence, our results also support that this effect is independent of the canonical functions of elastin in elastic fibers as they were not detected, and in elastokines, as they are only produced after fiber cleavage. In addition, the rapid molecular alterations, detected 24 h after siRNA transfection and the seeding of cells, also support elastin fiber- and elastokine-independent functions.

To gain further insights into this new molecular mechanism and this novel function of ELN, we performed non-biased transcriptomic analyses. Gene set enrichment analysis and differentially expressed gene analysis emphasized a sharp induction of HMOX1, revealing an unexpected link between *ELN*, *HMOX1* expression and heme metabolism. HMOX1 catalyzes heme degradation into three products: biliverdin, carbon monoxide and ferrous iron. All these components can display a broad range of beneficial or deleterious effects at the cellular level. For instance, biliverdin after transformation into bilirubin has ROS scavenging properties [48], carbon monoxide can exert cell protective effects [49] and iron can exert pro-oxidant effect as well as regulate the activity of many iron-dependent enzymes [34,35,50]. According to our results, upregulation of HMOX1 during *ELN* downregulation contributed to cellular senescence as HMOX1 knockdown decreased senescence. This is consistent with previous results supporting a pro-senescent role for HMOX1 [15]. Nevertheless, HMOX1 can also protect from cellular senescence [14,16], and in agreement with these results, HMOX1 knocked-down cells displayed a slight increase in senescence marks: *CDKN1A* level and SA-β-galactosidase activity. HMOX1 thus likely has a dual activity on senescence being pro-senescent when strongly activated, as observed here after *ELN* knockdown, and anti-senescent when expressed at a physiological level. This dual role could reflect the complexity of the role of the products of its enzymatic activity.

Even if iron was reported to increase in senescent cells [30,31] creating some novel opportunities for senolytic strategies [32], its potential functional role in regulating senescence was unknown until recently. Indeed it was recently reported that iron can induce cellular senescence and participates in senescence-dependent fibrotic lesions [51]. Our data, in the context of *ELN* downregulation-induced senescence, support that iron accumulation participates in senescence induction, as its chelation decreases *ELN* downregulation-induced cellular senescence, and also uncovers a non-yet explored mechanism explaining iron accumulation in senescent cells. Iron exerts multiple roles: it contributes to ROS production by the Fenton reaction, that could accentuate ROS production in *ELN*-downregulated cells, and it regulates the activity of a large family of oxygenases [34,35]. Epigenome modifications are critical regulators of cellular senescence [52,53] and a screening on iron-dependent epigenome modifiers highlighted a role for the PHF8 histone demethylase in the regulation of *ELN* knockdown-induced senescence suggesting that the iron increase is upstream of PHF8 during *ELN* downregulation-induced senescence. Although PHF8 has never been described to exert a senescence-regulatory role, other iron-dependent methylases, such as JMJD3/KDM6B, were described to regulate senescence [36,37], albeit none of these known senescence regulators significantly impacted *ELN* downregulation-induced senescence. Given the increase in iron observed in *ELN*-downregulated cells, other iron-dependent processes could also participate in the regulation of senescence. Defining the regions bound by PHF8 allowed the identification of many genes involved in the regulation of senescence upregulated by the loss of *ELN* and bound by PHF8, further supporting a role for PHF8 in regulating *ELN* knockdown-induced cellular senescence.

Overall, our work reveals an unexpected role for *ELN* in the regulation of senescence by modulating iron-dependent gene expression. This work paves the way for future research into this non-canonical role of *ELN* and its contribution to aging, to fully understand the mechanisms by which a decrease in *ELN* affects early production of ROS and upregulation of HMOX1, and whether targeting some of the herein described mechanisms (e.g., HMOX1, iron or PHF8) could be used to prevent and/or treat some age-related pathologies.

## 4 Methods and materials

### 4.1 Cell culture and reagents

MRC5 (ATCC, Manassas, VA, USA), 293 GP (virus-producing cells) (Clontech, Mountain View, CA, USA) and normal human diploid fibroblasts (NHDF, Biological Resource Center GCS/CTC) were cultured in Dulbecco's modified Eagle's medium (DMEM, Life Technologies, Carlsbad, USA) with GlutaMax and supplemented with 10% FBS (Sigma-Aldrich, Saint-Louis, USA) and 1% penicillin/streptomycin (ThermoFisher Scientific). MRC5 were treated with 72 nM deferoxamine (DFO) (D9533, Sigma-Aldrich) or 1 mM N-acetyl-cysteine (NAC) (A9165, Sigma-Aldrich) when indicated.

Mouse embryonic fibroblasts (MEFs) were generated from 10.5 to 12.5-day-old littermate embryos obtained from heterozygous *Eln* mouse intercrosses [54]. MEFs were cultured with DMEM with GlutaMax, 10% FBS, 1% penicillin/streptomycin, 1% of Gibco™ MEM Non-Essential Amino Acids Solution (ThermoFisher Scientific) and 1 mM NAC. Breeding and maintenance of *Eln* heterozygous mutant mice [54] for MEF preparation were approved by the French Minister of Research and Innovation in accordance with European Union Guidelines (ethical permit reference #25116–2020041515094224).

All cells were grown under standard conditions (37 °C, 5% CO_2_).

### 4.2 siRNA transfection

Cells were transfected with ON-Targetplus SMART pools of 4 siRNA targeting ELN, TP53, NRF2, HMOX1, PHF8 or iron-dependent demethylases, with individual siRNA targeting ELN (siELN #1 and #2) or with non-targeting siRNA pool as control (siCTRL) (Horizon Discovery). The list of sequences of individual siRNA is presented in Suppl. Table 2 and the sequences for the pools are available from the supplier. First, Dharmafect transfection reagent 1 (Horizon Discovery) was incubated with siRNA for 20 min in antibiotics and serum-free DMEM. Second, cells were reverse transfected in antibiotic-free, 10% FBS DMEM. Final concentration of siRNA was 15 nM. The following day, medium was changed with DMEM 10% FBS, 1% antibiotics.

### 4.3 Vectors, plasmid transfection and infection

pLPCx‐roGFP2‐ORP1, a retroviral vector encoding a ROS reporter previously described in [55], was used. 293 GP virus-producing cells were transfected with pLPCx‐roGFP2‐ORP1 using PEIpro transfection reagent (Polyplus) according to the manufacturer's recommendations. 48 h after transfection, viral supernatant was collected, diluted 1:4 with DMEM and hexadimethrine bromide (8 μg/mL; Sigma-Aldrich) was added. Viral supernatant was added to MRC5 cells, centrifuged at 2000 RPM for 30 min and then incubated for 8 h. One day after infection, selection was started using puromycin at 500 ng/ml.

### 4.4 Growth curves

At the beginning of the growth curve 200,000 cells were seeded in 10 cm^2^ dishes. Cells were counted and 200,000 cells were re-seeded at each passage. To calculate population doubling (PD), the following formula was used: PD = ln (N/N0)/ln2, N = number of cells; N0 = number of seeded cells.

### 4.5 Senescence-associated-β-galactosidase assay and crystal violet staining

Senescence-associated-β-galactosidase (SA-β-gal) activity was detected according to [56]. Cells were washed with 1X PBS, fixed with 2% formaldehyde and 0.2% glutaraldehyde in PBS for 5 min, washed twice with 1x PBS and incubated overnight at 37 °C in solution containing 1 mg/mL 5-bromo-4-chloro-3-indolyl-b-d-galactopyranoside, 5 mM potassium ferrocyanide, 5 mM potassium ferricyanide, 150 mM NaCl, 2 mM MgCl_2_, and 40 mM citric acid/Na phosphate buffer, pH 6.0. At least 100 cells were counted and the percentage of SA-β-gal-positive cells was calculated. For crystal violet staining, cells were washed with 1X PBS, fixed with 3,7 % formaldehyde for 15 min and then stained with crystal violet solution. Pictures of stained wells are presented.

### 4.6 RNA extraction, reverse transcription and real-time quantitative PCR

RNA was extracted with the use of Nucleozol according to the manufacturer's recommendations (Macherey-Nagel). Maxima First-Strand cDNA Synthesis Kit (ThermoFisher Scientific) was used to synthesize cDNA from 1 μg of RNA. Then cDNA was diluted and used as a template for quantitative PCR. qPCR mixture was prepared with 200 nM primers, SYBR™ Green PCR Master Mix (ThermoFisher Scientific) for the gene of interest. Relative mRNA levels were calculated using the Comparative Ct (ΔΔCT) method. mRNA levels of 2 housekeeping genes (GAPDH and HPRT1 for MRC5 and Gapdh and Tbp for MEFs) were used for normalization. Primer sequences used are listed in Suppl. Table 3.

### 4.7 Transcriptomic analysis

Transcriptomic analysis was performed using Whole Human Genome Oligo 4 × 44 K Microarrays (Agilent Technologies) and Agilent single-color gene expression workflow. First RNA was extracted using Nucleospin RNA (Macherey Nagel) and quality (RNA integrity number – RIN) was tested with 2200 TapeStation system (Agilent technology). cRNA was synthesized and labeled with Cy3 dye from 100 ng of total RNA using a single-color Low Input Quick Amp Labeling Kit (Agilent Technologies). Then, 1650 ng of Cy3-labeled cRNA purified with RNeasy Mini-spin columns (Qiagen) was hybridized on 4 × 44 K arrays for 17 h at 65 °C. The microarrays were rinsed and scanned using an Agilent G2565CA DNA microarray scanner (Agilent Technologies). Fluorescent signals were extracted using Feature Extraction Software Version 10.5.1.1 (Agilent Technologies) and then transferred to Genespring GX 12.6 software (Agilent Technologies) for data processing and data mining. Normalization was done on 75^th^ percentile intensity values, and baseline adjustment was done on control samples. Transcriptomic analysis was performed based on three independent biological replicates and differentially expressed genes were selected using moderated *t*-test p value < 0.01 and fold change cutoffs of 1.5-fold. Gene set enrichment analysis (GSEA) was performed using GSEA website (www.broadinstitute.org/gsea/). Pre-ranked GSEA was conducted on the ranked list of relative gene expression between sample versus control. Other visualizations were performed with ggplot2 and pheatmap packages in R studio.

### 4.8 Immunofluorescence

For analysis of fibers, normal human dermal fibroblasts (NHDF) and MRC5 cells were seeded onto 12 mm poly (lysine)-coated glass coverslips, and were grown for 8 days post-confluence in complete medium (DMEM, 10% FBS, 1 % penicillin/streptomycin) prior to fixation with 4 % paraformaldehyde. Cells were washed three times with PBS and blocked with PBS containing 5 % BSA and 10 % normal goat serum for 1 h at room temperature. Elastin, fibrillin-1 and type I collagen were immunodetected using mouse anti-elastin (MAB2503, Merk, 1/100), or rabbit anti-fibrillin-1 (HPA021057, Sigma-Aldrich, 1/500) or rabbit anti-type I collagen (20,111, Novotec, 1/100) antibodies in PBS containing 0.5 % BSA and 1 % normal goat serum at 4 °C overnight. Samples were washed three times with PBS and incubated with Alexa Fluor-546 goat anti-mouse (A11030, Invitrogen, 1/1000) or anti-rabbit (A11035, Invitrogen, 1/1000) IgG in PBS for 1 h at room temperature. Samples were washed three times with PBS and nuclei were counterstained with 1 μg/mL DAPI in PBS for 10 min at room temperature. Samples were washed three times with PBS, then coverslips were mounted on glass slides using Fluoromount-G (00-4958-02, Invitrogen). Pictures were acquired on an inverted confocal microscope Leica SP5.

For detection of HMOX1, cells were grown on 96-well plates (#6055300, PerkinElmer) and washed with 1x PBS. Then, cells were fixed with ice-cold methanol for 10 min, washed with 1x PBS and permeabilized with 0.5% Triton X-100 in PBS. Afterwards, cells were blocked with 3% bovine serum albumin (BSA), in PBS containing 0.1% Triton X-100 for 15 min. After washing, cells were incubated with primary anti-HMOX1 antibody (1:200) (sc-136,960, Santa Cruz) in blocking buffer for 2 h, and with Alexa 488 secondary antibody (1:500) (A11001, Life Technologies) in 1x PBS for 1 h. DNA was stained with Hoechst 33,342 (62,249, Life Technologies). Immunofluorescence was visualized with an Opera Phenix® Plus High-Content Screening System (PerkinElmer) and analyzed with Harmony High Content Imaging and Analysis software (PerkinElmer).

### 4.9 Mitochondrial ROS imaging and quantification

Cells stably expressing roGFP2‐ORP1 (redox-sensitive fluorescent protein) were rinsed and incubated in HBSS with calcium and magnesium (14025050, Thermo Scientific). Fluorescence was visualized with an Opera Phenix® Plus High-Content Screening System (PerkinElmer) using 405 and 488 nm for excitation and 500–554 nm for emission of the signal. Fluorescence intensity was quantified with Columbus software (PerkinElmer) and relative amount of oxidized/reduced roGFP was calculated.

### 4.10 Fe^2+^ imaging and quantification

Ferrous iron content of cells was determined by FerroFarRed also known as SiRhoNox-1 fluorescent probe based on manufacturers protocol (SCT037, Sigma-Aldrich). Briefly, cells were rinsed three times with HBSS buffer. Cells were then treated with 5 μM FerroFarRed diluted with serum-free cell culture medium and incubated for 1 h at 37 °C. After staining, excess probe was washed off with HBSS and fluorescence was visualized with an Opera Phenix® Plus High-Content Screening System (PerkinElmer) and quantified with FIJI software.

### 4.11 Iron measurements with ICP-MS

Ultrapure water (resistivity 18.2 MΩ.cm^−1^) was produced in a Millipore Synergy system (France). Concentrated technical grade HNO_3_ provided by Carlo Erba (France) was distilled at low temperature in Savillex PFA equipment. H_2_O_2_ 30% Suprapur was purchased from Merck (Germany). Single-element standard solutions from SCP Science (Québec, Canada) were used.

MRC5 cell pellets and cell culture media samples were digested in Savillex PFA beakers using 1 mL of HNO_3_ (14 M) and 0.2 mL of H_2_O_2_ (30%) at 120 °C for 48 h. Dissolved samples were then evaporated to dryness at 120 °C and redissolved in 5 mL of HNO_3_ (0.5 M). The Fe concentrations were measured using ICP-MS iCAP-Q, Thermo Scientific (Bremen, Germany). The measurements were carried out in a single collision cell mode, with kinetic energy discrimination (KED), using He as collision gas to avoid interferences on ^56^Fe ^+^ by ^40^Ar^16^O^+^. To correct the instrumental drift, In at 2 μg/L was used for the internal standard calibration.

### 4.12 PHF8 chromatin immunoprecipitation sequencing

MRC5 cells were fixed with freshly prepared formaldehyde solution (11% formaldehyde F-8775 Sigma-Aldrich, 0,1 M NaCl S3014 Sigma-Aldrich, 1 mM EDTA E5134 Sigma-Aldrich, 50 mM HEPES 54547 Sigma-Aldrich) added to the existing media for 15 min in room temperature. Next glycine solution (2,5 M glycine, G-7403, Sigma-Aldrich) was added for 5 min. Cells were scraped and centrifuged at 4 °C, 10 min, 800×*g*, then washed twice with ice cold PBS with Igepal (0,5%, I-8896, Sigma-Aldrich). PMSF (P-7626, Sigma-Aldrich) was added to ice cold PBS with Igepal in a final concentration of 1 mM. Then, the cells were pelleted again at 4 °C, 5 min, 800×*g* and snap-frozen on dry ice. ChIP seq was performed by Active Motif using PHF8 antibody (A301-772 A, Bethyl Laboratories).

### 4.13 Statistical analysis

Statistical analysis and graphs, presented as mean of three or more independent experiments with SEM, were created with GraphPad Prism 9. Parametric tests were two-tailed, unpaired or paired: Student's *t*-test (equal variance) or Welch's *t*-test (for non-equal variance). For non-parametric test, Mann–Whitney *U* Test was performed. For multiple comparisons (>2), one- or two-way ANOVA and then paired or unpaired Tukey's multiple comparisons test were performed.

## CRediT authorship contribution statement

**Joanna Czarnecka-Herok:** Writing – original draft, Visualization, Validation, Methodology, Investigation, Formal analysis, Conceptualization. **Kexin Zhu:** Writing – review & editing, Visualization, Methodology, Investigation. **Jean-Michel Flaman:** Writing – review & editing, Visualization, Resources, Methodology, Investigation, Formal analysis, Data curation. **Delphine Goehrig:** Writing – review & editing, Resources, Methodology. **Mathieu Vernier:** Writing – review & editing, Visualization, Methodology, Formal analysis. **Gabriela Makulyte:** Writing – review & editing, Resources, Methodology. **Aline Lamboux:** Writing – review & editing, Methodology. **Helena Dragic:** Writing – review & editing, Methodology. **Muriel Rhinn:** Writing – review & editing, Methodology, Investigation. **Jean-Jacques Médard:** Writing – review & editing, Methodology. **Gilles Faury:** Writing – review & editing, Resources. **Philippe Bertolino:** Writing – review & editing, Resources, Methodology. **Vincent Balter:** Writing – review & editing, Resources, Methodology. **Romain Debret:** Writing – review & editing, Methodology, Funding acquisition, Conceptualization. **Serge Adnot:** Writing – review & editing, Visualization, Funding acquisition, Conceptualization. **Nadine Martin:** Writing – original draft, Visualization, Supervision, Project administration, Methodology, Investigation, Funding acquisition, Formal analysis, Conceptualization. **David Bernard:** Writing – original draft, Validation, Supervision, Project administration, Funding acquisition, Formal analysis, Conceptualization.

## Declaration of competing interest

The authors have competing interests to declare.

## Data Availability

Transcriptomic data are available in GEO GSE268042. Other data will be made available on request.
