# Supplementary material for: A non-canonical role of ELN protects from cellular senescence by limiting iron-dependent regulation of gene expression
Source: Redox Biol. 2024 May 22;73:103204. doi: 10.1016/j.redox.2024.103204 (PMC11167390; doi:10.1016/j.redox.2024.103204)
Supplement: Multimedia component 1 [file mmc1.pdf]

SUPPLEMENTARY FIGURE LEGENDS

Supplementary Figure 1.

A. RT-qPCR of *ELN* gene 4 days after transfection of MRC5 cells with non-targeting siCTRL or two individual siRNAs targeting ELN (siELN #1 and siELN #2). Mean +/- SEM of n=3 independent experiments. Unpaired two-tailed Welch's t-test. **B.** Crystal violet staining (top panel) and quantification of cell number (lower panel) 7 days after transfection of MRC5 fibroblasts with siCTRL or two individual siELN (#1 and #2). Top panel: representative experiment (n=3). Lower panel: mean +/- SEM of n=3 independent experiments, unpaired two-tailed Welch's t-tests. **C**. Representative micrographs (left panel) and quantification (right panel) of SA-β-gal-positive cells in WT and *Eln* KO mouse embryonic fibroblasts. Mean +/- SEM of n=4-5 independent embryos. Unpaired two-tailed Welch's t-test. **D.** Quantification of SA-β-gal-positive cells 4 days after transfection of MRC5 with siCTRL or two individual siELN. Mean +/- SEM of n=3 independent experiments. Unpaired two-tailed Welch's t-test **E**. RT-qPCR of *CDKN1A* gene 4 days after transfection of MRC5 cells with non-targeting siCTRL or individual siRNAs targeting ELN (siELN #1 and siELN #2). Mean +/- SEM of n=3 independent experiments. Unpaired two-tailed Welch's t-test. **F**. RT-qPCR of *GDF15*, *MMP3*, *BMP2* and *ANGPTL4* genes at day 4 after transfection of MRC5 fibroblasts with siCTRL or siELN pools. Mean +/- SEM of n=4 independent experiments. Unpaired two-tailed Welch's t-test. **G**. Volcano plot showing differentially expressed genes in siELN *versus* siCTRL MRC5 fibroblasts 4 days after siRNA transfection, according to transcriptomic analyses (n=3). **H.** Immunofluorescence micrographs of normal human dermal fibroblasts (NHDF) and MRC5 normal human embryonic lung fibroblasts showing staining of elastin, fibrillin-1 and collagen I as indicated (in red) and of nuclei with DAPI (in blue) (representative pictures of n=2).

Supplementary Figure 2.

A. Heatmap showing the profile of expression of the 13 genes from GSEA Reactome gene set "heme signaling" enriched by ELN knockdown (4 days after siRNA transfection). **B.** RT-qPCR of *ELN* and *HMOX1* genes 2 days after transfection of MRC5 cells with siCTRL, siELN and/or siHMOX1 as indicated. Mean +/- SEM of n=4 independent experiments. Two-way ANOVA. Tukey's multiple comparisons test (left panel). Unpaired two-tailed Welch's t-test (right panel).

Supplementary Figure 3.

A. RT-qPCR of *ELN* and *TP53* genes 2 days after siRNA transfection of MRC5 cells with siCTRL, siELN and/or siTP53 as indicated. Mean +/- SEM of n=4 independent experiments. One-way ANOVA. Tukey's multiple comparisons test. **B.** RT-qPCR of *ELN* and *NRF2* genes 2 days after transfection of MRC5 cells with siCTRL, siELN and/or siNRF2 as indicated. Mean +/- SEM of n=4 independent experiments. One-way ANOVA. Tukey's multiple comparisons test. **C.** Cell number quantification 7 days after the indicated siRNA transfection of MRC5 cells. Representative experiment (n=4). **D.** Quantification of SA-β-gal-positive cells 4 days after the indicated siRNA transfection of MRC5 cells. Mean +/- SEM of n=4 independent experiments. One-way ANOVA. Tukey's multiple comparisons test. **E.** Quantification of cell number 4 days after the indicated siRNA transfection of MRC5 cells. Mean +/- SEM of n=3 independent experiments. One-way ANOVA. Tukey's multiple comparisons test. **F.** Quantification of SA-β-gal-positive cells 4 days after the indicated siRNA transfection of MRC5 cells. Mean +/- SEM of n=3 independent experiments. One-way ANOVA. Tukey's multiple comparisons test. **G.** RT-qPCR of *ELN* gene 4 days after siRNA transfection of MRC5 cells and 1 mM NAC treatment where

indicated. Mean +/- SEM of n=4 independent experiments. One-way ANOVA. Tukey's multiple comparisons test.

Supplementary Figure 4.

RT-qPCR of *ELN* gene 4 days after siRNA transfection of MRC5 cells and DFO treatment where indicated. Mean +/- SEM of n=4 independent experiments. One-way ANOVA.

Supplementary Figure 5.

A. RT-qPCR of *ELN* and *PHF8* genes 4 days after transfection of MRC5 cells with siCTRL, siELN and/or siPHF8. Mean +/- SEM of n=3 independent experiments. One-way ANOVA. Tukey's multiple comparisons test. **B**. Pie chart of PH8 ChIP-seq data in MRC5 in siCTRL and siELN conditions. **C.** Promoters average plot generated from PHF8 ChIP-seq data in siCTRL- or siELN-transfected MRC5. **D.** Venn diagram showing the number of genes shared between genes bound by PHF8 and upregulated by ELN knockdown 1 day after siRNA transfection and genes of the CellAge signature. Fisher's exact test P value is indicated for CellAge signature enrichment.

Suppl. Fig. 1

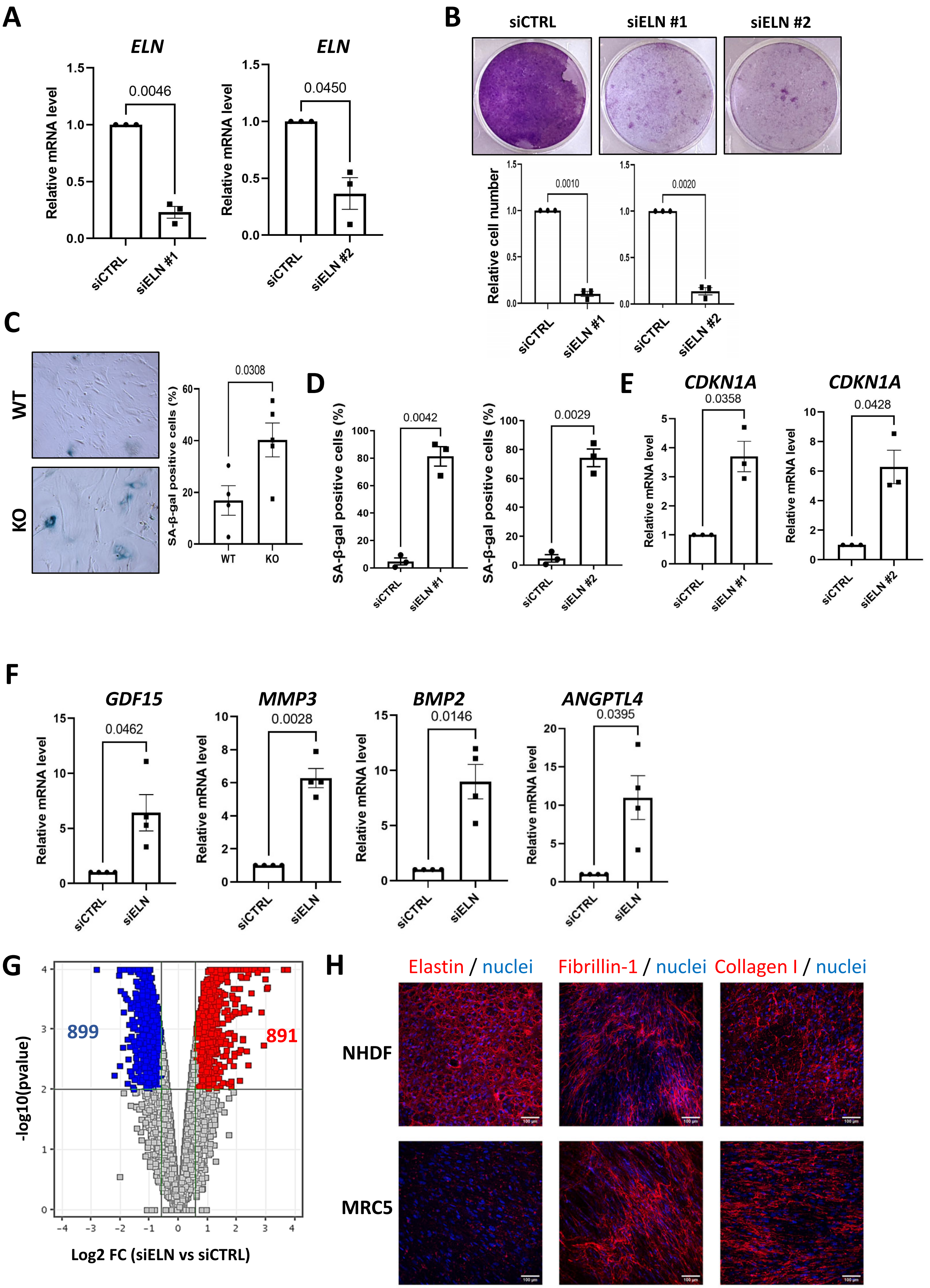

A
ELN
Relative mRNA level
0.0046
siCTRL
siELN #1
ELN
0.0450
siELN #2
B
siCTRL
siELN #1
siELN #2
Relative cell number
0.0010
0.0020
C
WT
KO
SA-β-gal positive cells (%)
0.0308
D
0.0042
0.0029
E
CDKN1A
0.0358
CDKN1A
0.0428
F
GDF15
0.0462
MMP3
0.0028
BMP2
0.0146
ANGPTL4
0.0395
siELN
G
899
891
-log10(pvalue)
Log2 FC (siELN vs siCTRL)
H
Elastin / nuclei
Fibrillin-1 / nuclei
Collagen I / nuclei
NHDF
MRC5

Suppl. Fig. 2

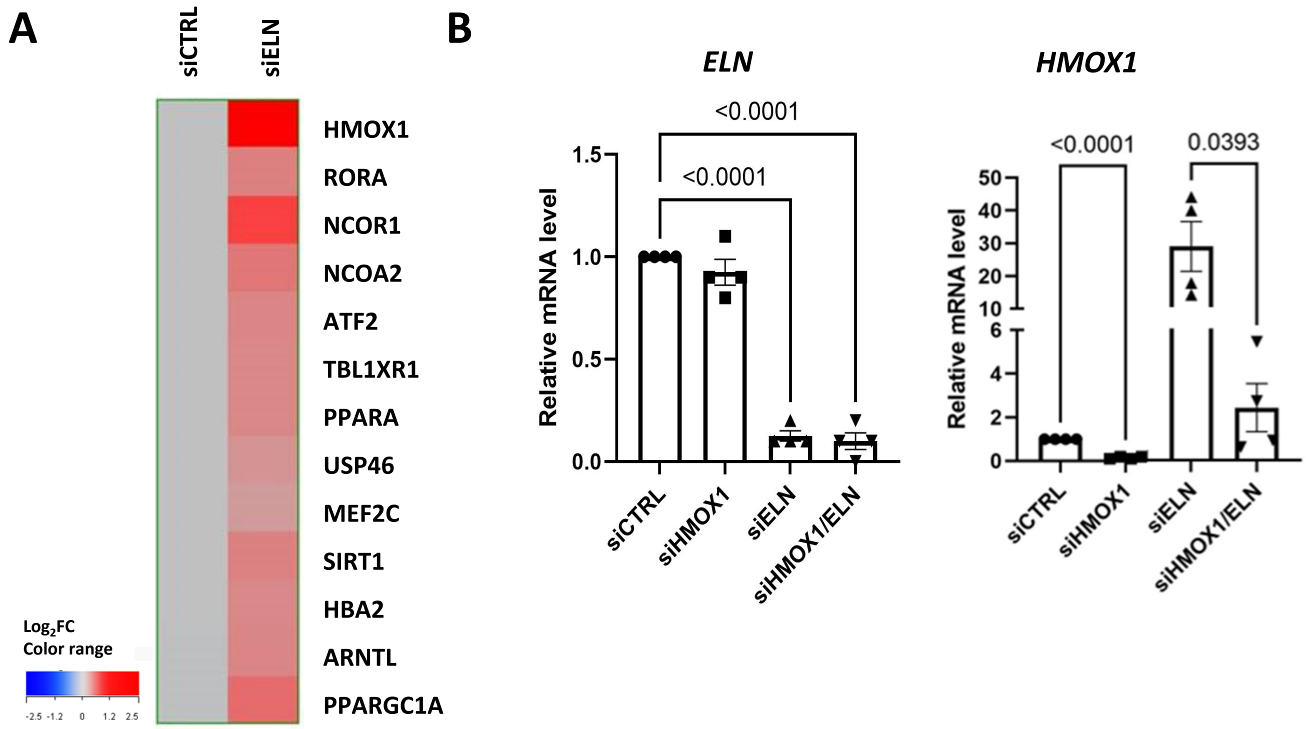
A
siCTRL
siELN
HMOX1
RORA
NCOR1
NCOA2
ATF2
TBL1XR1
PPARA
USP46
MEF2C
SIRT1
HBA2
ARNTL
PPARGC1A
Log2FC
Color range
-2.5 -1.2 0 1.2 2.5
B
ELN
<0.0001
<0.0001
Relative mRNA level
1.5
1.0
0.5
0.0
siCTRL
siHMOX1
siELN
siHMOX1/ELN
HMOX1
<0.0001
0.0393
Relative mRNA level
50
40
30
20
10
6
4
2
0
siCTRL
siHMOX1
siELN
siHMOX1/ELN

Suppl. Fig. 3

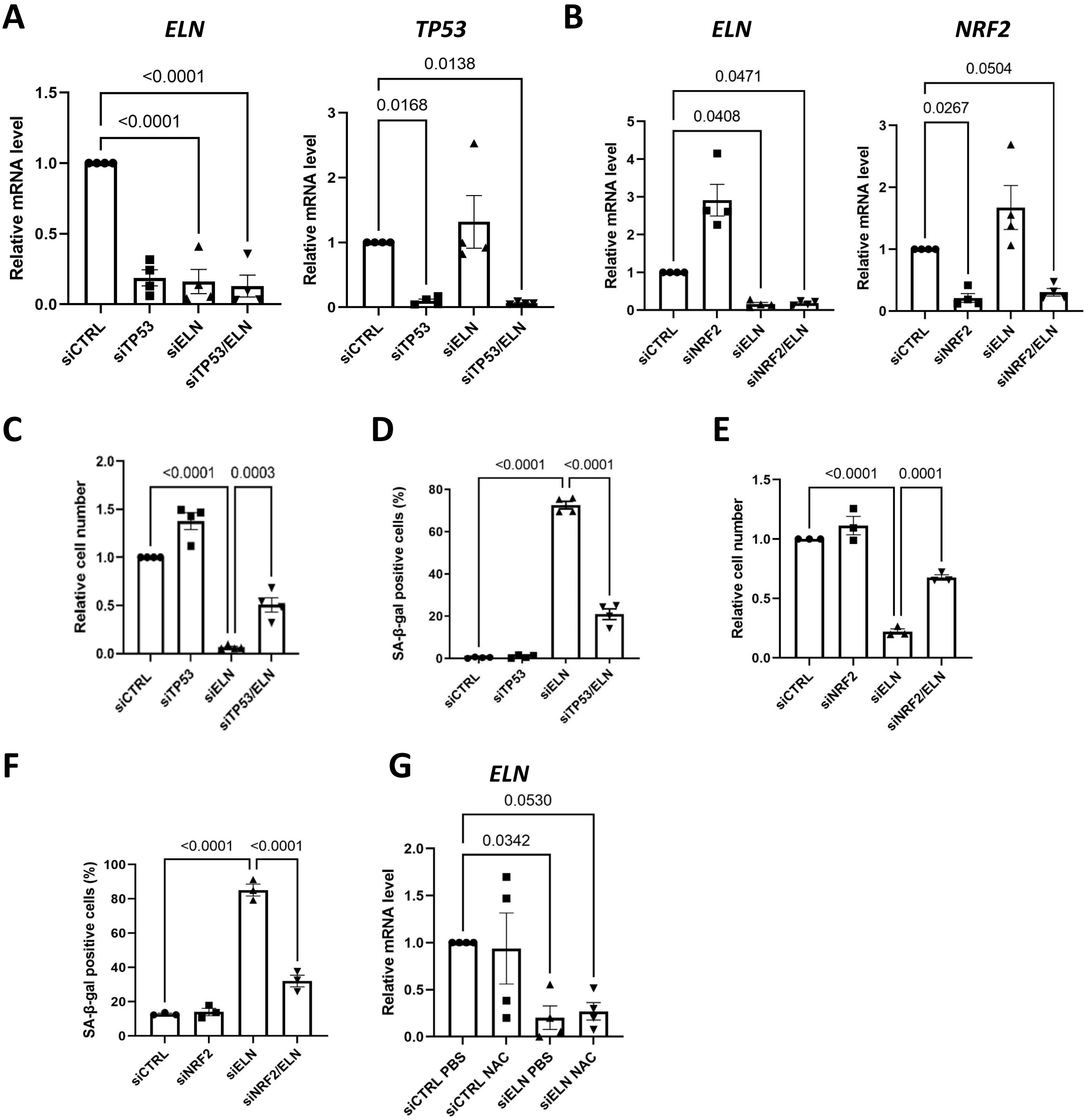

A
ELN
Relative mRNA level
<0.0001
<0.0001
siCTRL
siTP53
siELN
siTP53/ELN
TP53
0.0138
0.0168
B
ELN
0.0471
0.0408
siNRF2
siNRF2/ELN
NRF2
0.0504
0.0267
C
Relative cell number
<0.0001
0.0003
D
SA-β-gal positive cells (%)
<0.0001
<0.0001
E
<0.0001
0.0001
F
<0.0001
<0.0001
G
ELN
0.0530
0.0342
siCTRL PBS
siCTRL NAC
siELN PBS
siELN NAC

Suppl. Fig. 4

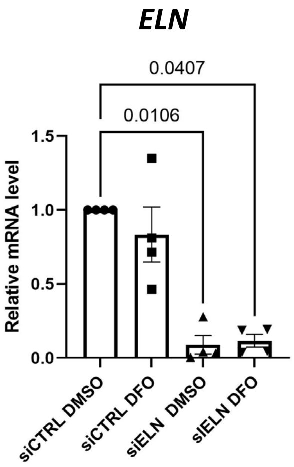
ELN
0.0407
0.0106
Relative mRNA level
1.5
1.0
0.5
0.0
siCTRL DMSO
siCTRL DFO
siELN DMSO
siELN DFO

Suppl. Fig. 5

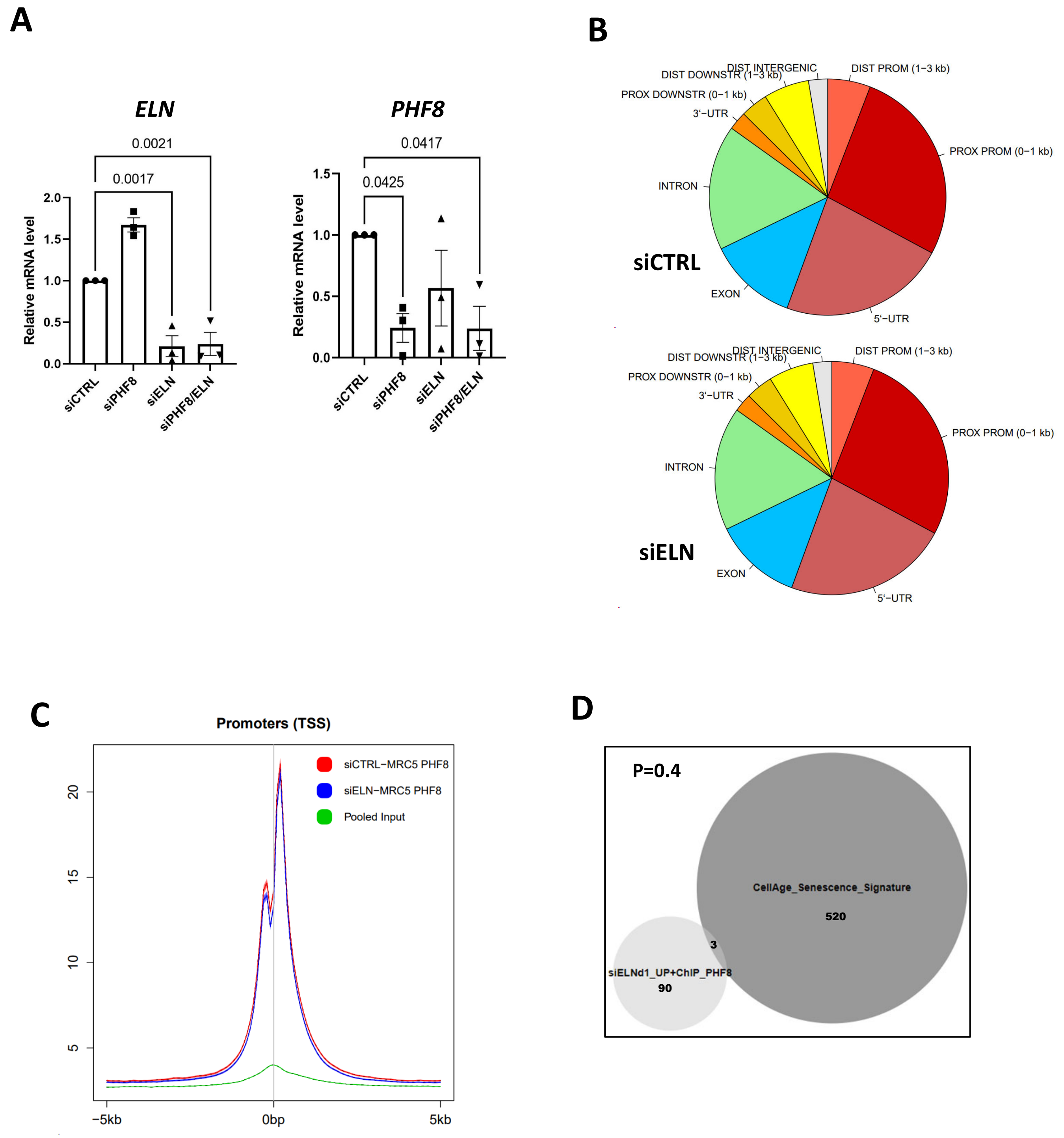
A
ELN
0.0021
0.0017
Relative mRNA level
2.0
1.5
1.0
0.5
0.0
siCTRL
siPHF8
siELN
siPHF8/ELN
PHF8
0.0417
0.0425
Relative mRNA level
1.5
1.0
0.5
0.0
siCTRL
siPHF8
siELN
siPHF8/ELN
B
DIST INTERGENIC
DIST DOWNSTR (1−3 kb)
DIST PROM (1−3 kb)
PROX DOWNSTR (0−1 kb)
3'−UTR
PROX PROM (0−1 kb)
INTRON
siCTRL
EXON
5'−UTR
DIST INTERGENIC
DIST DOWNSTR (1−3 kb)
DIST PROM (1−3 kb)
PROX DOWNSTR (0−1 kb)
3'−UTR
PROX PROM (0−1 kb)
INTRON
siELN
EXON
5'−UTR
C
Promoters (TSS)
siCTRL−MRC5 PHF8
siELN−MRC5 PHF8
Pooled Input
20
15
10
5
−5kb
0bp
5kb
D
P=0.4
CellAge_Senescence_Signature
520
3
siELNd1_UP+ChIP_PHF8
90

Suppl. Table 1

93 genes bound by PHF8 (ChIP) and up-regulated one day after *ELN* knockdown		389 genes bound by PHF8 (ChIP) and upregulated 4 days after *ELN* knockdown							
ACVR2A	PDGFA	PLXNB1	HSD11B1L	PGAP3	MFSD9	N4BP2L2	NUDT16L1	RASSF7	TMEM134
ADAT3	PEX11A	MAP1LC3B	C15orf65	MAP3K13	PLOD1	CCDC107	DHRS7	IGFBP7	SERTAD1
ANKEF1	PGPEP1	WDR37	COL5A1	WIPI2	PSMG3-AS1	ABTB2	MSC	HHAT	FNDC4
ANKRD46	POMK	PLA2G15	CDC34	PPME1	VPS53	ATP6AP1	EPHX4	AGTRAP	C1orf229
AP1S2	RBM22	FAXDC2	ADAMTSL4	SRRM2-AS1	THBS1	NDFIP1	RABGAP1L	CLCF1	HOXA4
AQP11	RBM33	TRPM4	CLSTN3	MANBA	PROSER2	ZNF425	CD274	MLPH	LRRC24
ARHGEF10	RHPN2	MPZL1	LYPD5	ACP2	HBEGF	BMP1	PLD3	DDRGK1	BAK1
ARRDC3	RIMS3	FLYWCH1	RGS2	SMIM13	FRY	TIMP1	ZMAT3	PTPRJ	CYB5R1
ATXN1	RNF19B	MMP14	BPGM	HAGH	SRRM3	SLC17A5	LRP10	UNC5B-AS1	ABHD17A
AVL9	RPS6KL1	ZNF136	LCOR	TM7SF3	NRP2	GDE1	PROS1	NAAA	B3GAT3
BBC3	SERAC1	LRRC73	TCTA	IL4R	SNAI1	FUCA1	CCNT2-AS1	NCKAP5L	NECAB3
BRWD1	SESN1	MAGIX	MFSD12	ITGA1	ITGA5	TM7SF2	PINK1	SLC43A2	RAB3A
CAMKMT	SLC43A2	DDA1	TRIB2	ULK1	HM13	ATP7B	CCPG1	TST	SPSB3
CCNJ	SPRED2	B3GALT4	MT1X	TOR4A	EMC10	SERPINB8	CACNA2D1	ITGB5	NRBP2
CPEB2	TDRKH	CHPF2	FANK1	WDR78	PSTPIP2	LPCAT2	ADGRE5	PICK1	PIDD1
CPEB3	TMEM170B	IQCE	GDPD5	MICA	DCBLD2	DNAJC1	KDM5C	KDM7A	HSD3B7
DCBLD2	TMEM64	DBNDD1	TP53INP2	BTG2	ANGPT1	FADS3	ANTXR1	MSC-AS1	LAMB2
FGFR1OP2	TMPO	TM9SF2	RHBDD2	ABCC3	EPB41L5	CDH6	TMEM8B	NRK	COX7A1
FZD5	TRIB2	FKBP1B	TMEM170B	MED14OS	ZNF841	GGT7	TFPI2	PAPPA	TFPT
GAB1	TTLL7	EME2	NPDC1	ARHGEF37	ALAS1	PLAT	TMEM59	PLAUR	GLMP
HACD2	VEGFA	MIEF2	AP5Z1	EXTL1	LGMN	TECPR2	LIN7B	MPC2	LZTS3
HELB	ZBTB26	SMAD7	PACS2	ABLIM3	SEMA6A	SEC14L1	IKBIP	PMEPA1	TSGA10
HERC6	ZBTB34	COLEC10	YPEL3	BAIAP2	ATP6V1G1	SPRY4	MAPRE3	STEAP3	BAD
HMBOX1	ZBTB37	FBXL19	SYNGR1	RHOB	KBTBD8	LRRC1	SPRYD3	SNCA	ENG
HMOX1	ZC3H14	SERPINI1	NKIRAS2	PTGER1	TGFBI	LRRC17	LYPLA2	ZMYM3	FAM219B
HNRNPA3	ZC3H8	FBXO36	PTP4A3	CACFD1	ZNF836	ADAMTS12	N4BP1	SLC2A11	LTBP3
IPMK	ZFP3	PPP1R3F	GSTZ1	GLS	KCNG1	CDIPT	TEN1	HIVEP1	GBA
ITGB8	ZNF138	KCNAB2	TMEM106A	PLK3	ARRDC1	COL4A5	BCAP31	PTPRK	ZNF837
KBTBD8	ZNF141	NOXA1	STXBP1	ADAMTS5	CREG1	PXDC1	FSTL3	ADAMTS10	CCT6B
KLHL15	ZNF214	HMGA2	MSL3	ARRDC4	PTGS2	ANGPTL2	TSPAN12	ID3	ZNF432
L2HGDH	ZNF253	MUC1	ZNF582-AS1	HMCN1	CD9	ITGB3	BACE1	TGM2	TMEM234
LAMA4	ZNF267	LEPR	MLLT11	CRELD1	FBXL18	LRPAP1	ADAMTS2	KLF9	TMBIM1
LATS1	ZNF318	UBTD1	ULBP2	BRF2	ZNF566	RPS6KL1	CD82	APBA1	ZNF582
LCOR	ZNF461	MXD1	GRK4	WDR26	SCN3B	GLB1	MIB2	TMEM191A	WDR45
LONRF1	ZNF501	ZBTB47	ERGIC1	EMC7	ANGPTL4	TMED1	EXD3	ATXN1	SLC12A4
LRRC8B	ZNF550	PARD6B	OSCP1	ZNF792	DUSP23	NEU1	PTGS1	SVIL	SEZ6L2
MAP3K8	ZNF569	QPCTL	ANKRD42	IL17RA	PRRG1	DYNLT3	LOC10013131<br>5	HSPB8	ECE1
MESP1	ZNF680	CDK13	CFAP57	PHLDA3	BVES	MAN2A2	TOM1	MFSD3	ABCA3
MFSD9	ZNF696	ANKRD11	NEK11	PTP4A1	P4HA2	CTSZ	MFSD1	SMAD5-AS1	SCARB1
NAMPT	ZNF736	DOK6	STX17-AS1	TIGAR	SLC22A5	NDST2	MRAS	WLS	NPC1
NCR3LG1	ZNF737	TSPAN14	IDUA	JUP	ZNF396	CD276	TNFRSF10D	CRIP2	CCND3
NFIX	ZNF792	MAP3K10	ZNF667-AS1	LIMK2	TGFB2	MT1E	MMP2	IL6	LGR4
NKIRAS1	ZNRF3	MMP16	DNAJB9	KIF16B	PGPEP1	CTSD	NME3	TFEB	SLC20A2
NSD1		COL4A6	ITGA2	ERV3-1	PPIC	TMEM87B	CTSL	CDKN1A	TOM1L2
PARD6B		PPP2R5B	RUSC2	C1S	ANKRD46	ABCC5	P4HTM	COL9A2	FBXL13
PARG		SNAI3-AS1	TMEM200A	ID1	PSAP	YPEL5	NNMT	GDNF	SYNE1
PARP4		PLXDC2	KCTD16	SGSM3	TMEM63A	APCDD1L	HSPB7	LYPD1	
PARP9		ANKH	RIC1	NCOA3	CALCOCO1	SECISBP2L	ADAM19	HOXB2	
PCGF5		CHAC1	ZFHX2	PCDH9	ATP6V0A1	C14orf28	KRT18	NMB	

Suppl. Table 1. List of genes bound by PHF8 and upregulated at different times after *ELN* knockdown.

Suppl. Table 2

Targeted gene	Catalog number	Sequence
ELN Individual #1	J-009306-07	CCGCUAAGGCAGCCAAGUA
ELN Individual #2	J-009306-08	CCGCCAAGGCUGCCAAGUA

Suppl. Table 2. List of individual siRNA sequences used for siRNA transfection.

Suppl. Table 3

HUMAN	Forward	Reverse
ELN	GTGCTGGTGTTCCTGGACTT	GCCAGGGCTCCAGGTACT
TP53	AGGCCTTGGAACTAAGGAT	CCCTTTTTGGACTTCAGGTG
HMOX1	GGCAGAGGGTGATAGAAGA	AGCTCCTGCAACTCCTCAAA
HPRT1	TGACCTTGATTTATTTTGCATACC	CGAGCAAGACGTTCAGTCCT
GAPDH	AGCCACATCGCTCAGACAC	GCCCAATACGACCAAATCC
CDKN1A	TCACTGTCTTGTACCCTTGTGC	GGCGTTTGGAGTGGTAGAAAT
BMP2	CGGACTGCGGTCTCCTAA	GGAAGCAGCAACGCTAGAAG
ANGPTL4	GACAAGAACTGCGCCAAGA	GCCGTTGAGGTTGGAATG
GDF15	CCGGATACTCACGCCAGA	AGAGATACGCAGGTGCAGGT
MMP3	GCAGTTTGCTCAGCCTATCC	TTTCTCCTAACAAACTGTTTCACATC
PHF8	CTCGCCATCATTCACTGT	GGTCACATCAATCACATCAATC
NRF2	AGACGGTATGCAACAGGACA	TTGTCAACTTCTGTCAGTTTGGC
MURINE	Forward	Reverse
Eln	GCTGCTGCTAAGGCTGCTAA	AGCACCTGGGAGCCTAACTC
Hmox1	ACATGGCCTTCTGGTATGG	CTCGTGGAGACGCTTTACATAG
Gapdh	CCTGCTTCACCACCTTCTTG	TGTCCGTCGTGGATCTGAC
Cdkn1a	TGAGCTGCGCCAGCTGAGGTGTG	AACATCTCAGGGCCGAAA
Tbp	GGGGAGCTGTGATGTGAAGT	CCAGGAAATAATTCTGGCTCA

Suppl. Table 3. List of human and murine primer sequences used for RT-qPCR.
